# Previous reproductive success and environmental variation influence nest‐site fidelity of a subarctic‐nesting goose

**DOI:** 10.1002/ece3.70313

**Published:** 2024-10-11

**Authors:** Jordan M. Thompson, Brian D. Uher‐Koch, Bryan L. Daniels, Thomas V. Riecke, Joel A. Schmutz, Benjamin S. Sedinger

**Affiliations:** ^1^ College of Natural Resources University of Wisconsin‐Stevens Point Stevens Point Wisconsin USA; ^2^ U.S. Geological Survey Alaska Science Center Anchorage Alaska USA; ^3^ Yukon Delta National Wildlife Refuge U.S. Fish and Wildlife Service Bethel Alaska USA; ^4^ Wildlife Biology Program University of Montana Missoula Montana USA

**Keywords:** *Anser canagicus*, emperor goose, nest survival, nest‐site fidelity, win‐stay lose‐switch

## Abstract

Nest‐site fidelity is a common strategy in birds and is believed to be adaptive due to familiarity with local conditions. Returning to previously successful nest sites (i.e., the win‐stay lose‐switch strategy) may be beneficial when habitat quality is spatially variable and temporally predictable; however, changes in environmental conditions may constrain dispersal decisions despite previous reproductive success. We used long‐term (2000–2017) capture‐mark‐reencounter data and hierarchical models to examine fine‐scale nest‐site fidelity of emperor geese (*Anser canagicus*) on the Yukon–Kuskokwim Delta in Alaska. Our objectives were to quantify nest‐site dispersal distances, determine whether dispersal distance is affected by previous nest fate, spring timing, or major flooding events on the study area, and determine if nest‐site fidelity is adaptive in that it leads to higher nest survival. Consistent with the win‐stay lose‐switch strategy, expected dispersal distance for individuals that failed their nesting attempt in the previous year was greater (207.7 m, 95% HPDI: 151.1–272.7) than expected dispersal distance for individuals that nested successfully in the previous year (125.5 m, 95% HPDI: 107.1–144.9). Expected dispersal distance was slightly greater following years of major flooding events for individuals that nested successfully, although this pattern was not observed for individuals that failed their nesting attempt. We did not find evidence that expected dispersal distance was influenced by spring timing. Importantly, dispersal distance was positively related to daily survival probability of emperor goose nests for individuals that failed their previous nesting attempt, suggesting an adaptive benefit to the win‐stay lose‐switch strategy. Our results highlight the importance of previous experience and environmental variation for informing dispersal decisions of a long‐lived goose species. However, it is unclear if dispersal decisions based on previous experience will continue to be adaptive as variability in environmental conditions increases in northern breeding areas.

## Graphical abstract

We found that nest‐site dispersal decisions of emperor geese in western Alaska follow the win‐stay lose‐switch strategy, and that this is adaptive in that it leads to higher nest survival. We also found that nest‐site dispersal distance is influenced by the occurrence of major flooding events in the previous year, but is not influenced by spring timing. Our results highlight the importance of previous experience and environmental variation in shaping dispersal decisions of a long‐lived goose species.

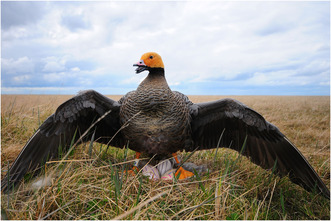

## 1 INTRODUCTION

Evolutionary theory predicts that decisions made by animals, such as selection of breeding habitat, should ultimately lead to an increase in fitness (Clark & Shutler, 1999; Martin, 1998; Piper, 2011). This applies especially to birds, as their reproductive success is primarily influenced by predation (Ricklefs, 1969), and individual decisions on where to nest can affect the risk of predation (Davis, 2005; Martin & Roper, 1988). Nest‐site fidelity, defined here as the tendency to return to a previous nest site, is a common strategy in birds and is believed to be beneficial due to familiarity with local resources, threats, and neighbors (Anderson et al., 1992; Greenwood & Harvey, 1982; Hinde, 1956), although empirical evidence for adaptive benefits of site familiarity is limited (Piper, 2011). Whether nest‐site fidelity is adaptive often depends on spatial and temporal variation in habitat quality (Switzer, 1993). When habitat quality is spatially variable but temporally predictable, dispersal decisions based on previous reproductive outcomes should be favored (i.e., the win‐stay lose‐switch strategy; Schmidt, 2004). The win‐stay lose‐switch strategy predicts that an individual will be more likely to return to the same nest site if they were previously successful at that site, and more likely to disperse if they were not successful. Alternative strategies, such as fidelity regardless of previous reproductive outcome, may be favored when habitat quality is spatially homogeneous but temporally variable (i.e., predictability of reproductive outcomes between years is low; Gerber et al., 2019; Switzer, 1993).

Nest‐site fidelity has been studied extensively in waterfowl (Anatidae). Empirical evidence often suggests use of the win‐stay lose‐switch strategy in nest‐site fidelity decisions of cavity‐nesting waterfowl (Dow & Fredga, 1983; Gauthier, 1990; Hepp & Kennamer, 1992); however, evidence for the win‐stay lose‐switch strategy has been variable in ground‐nesting waterfowl, with minimal or no evidence in some studies (Bustnes & Erikstad, 1993; Lecomte, Gauthier, et al., 2008) and support in others (Fowler, 2005; Lindberg & Sedinger, 1997; Öst et al., 2011). Furthermore, observed fitness consequences of nest‐site fidelity in waterfowl are variable, and have included greater clutch sizes (Gauthier, 1990; Lindberg & Sedinger, 1997), earlier nest initiation dates (Hepp & Kennamer, 1992; Öst et al., 2011), and higher nest survival (Fowler, 2005), with some species exhibiting all of these (e.g., common goldeneyes *Bucephala clangula*; Dow & Fredga, 1983).

Several studies have examined nest‐site fidelity in Arctic‐ and subarctic‐nesting geese, with mixed support for the win‐stay lose‐switch strategy and associated fitness consequences of nest‐site fidelity (Fowler, 2005; Lecomte, Gauthier, et al., 2008; Lindberg & Sedinger, 1997). Timing of nesting for Arctic‐ and subarctic‐nesting geese is complicated by short growing seasons, where hatch is timed to coincide with peak forage quality of food plants (Owen, 1980; Sedinger & Raveling, 1986). This often causes timing of nesting to coincide with spring timing (Ely & Raveling, 1984; Petersen, 1990). Delayed springs may lead to a longer interval between the time geese arrive on the breeding areas and initiate their nests (Hupp et al., 2006) or cause geese to initiate nests while there is still a high percentage of snow cover on the landscape (Petersen, 1990). Thus, geese may be faced with trade‐offs between timing of nesting and fidelity to a previous nest site in late springs when previous nest sites may not be available, making dispersal more likely in years of late spring onset (Lecomte, Gauthier, et al., 2008). The onset of spring in Arctic and subarctic breeding areas is advancing and becoming more variable with changes in temperature and precipitation associated with climate change (Box et al., 2019; Schmidt et al., 2023). Progressively earlier springs may allow geese to consistently return to nest sites where they were previously successful. The interplay between environmental conditions, nest‐site fidelity, and its associated fitness consequences are important to understand given rapid environmental change (Kloskowski, 2021; Merkle et al., 2022), although these topics have been relatively understudied in this context.

**FIGURE 1:**
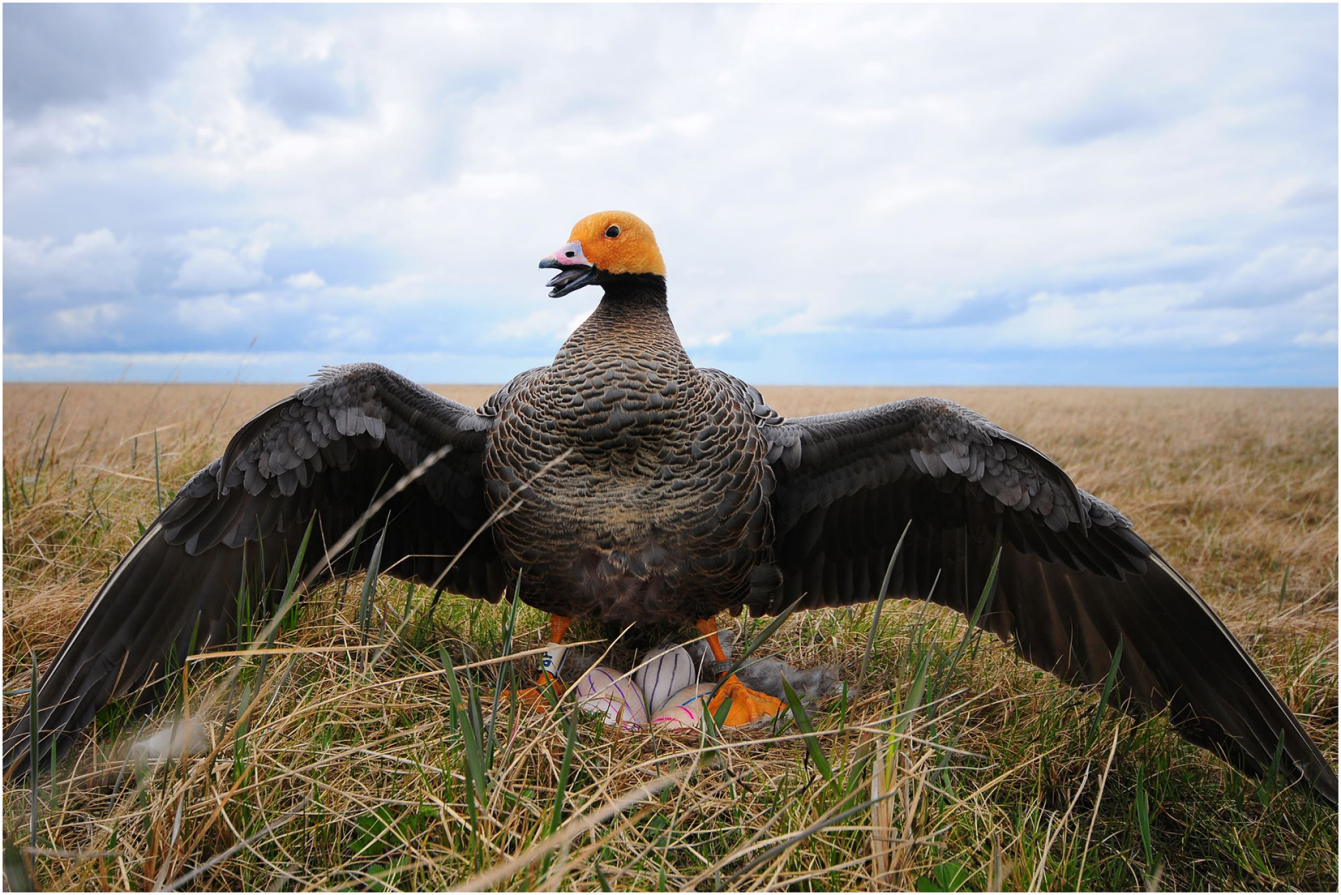
Photograph of a banded emperor goose (*Anser canagicus*) at the Manokinak River study site on the Yukon–Kuskokwim Delta in western Alaska. The photograph was taken by Ryan Askren, U.S. Geological Survey.

We used 17 years of capture‐mark‐reencounter data for emperor geese (*Anser canagicus*; Figure 1) at a breeding site on the Yukon–Kuskokwim Delta in western Alaska (Thompson & Uher‐Koch, 2024) to test the win‐stay lose‐switch strategy by quantifying fine‐scale nest‐site dispersal distances and examining whether dispersal distance was influenced by previous nest fate, spring timing, or major flooding events. We also tested whether the win‐stay lose‐switch strategy was adaptive in that it led to higher nest survival (i.e., the probability that at least one egg in a nest hatches). Consistent with the win‐stay lose‐switch strategy, we predicted that mean nest‐site dispersal distance would be greater for birds that failed their previous nesting attempt than for those that nested successfully. We predicted that dispersal distance would be higher in years with relatively late springs as greater snow cover and melt water might limit availability of nest sites in those years and force individuals to move farther distances from their previous nest site. Furthermore, we predicted that dispersal distance would be greater following years with major flooding events due to much lower nest survival in those years (Thompson et al., 2023) and potential for brood mortality during floods. Lastly, we predicted that moving a greater distance following a previous nest failure would result in greater nest survival.

## 2 MATERIALS AND METHODS

### 2.1 Study species and data collection

The emperor goose is a long‐lived marine species endemic to the Bering Sea region (Eisenhauer & Kirkpatrick, 1977). Emperor geese nest primarily on the Yukon–Kuskokwim Delta in western Alaska, but small numbers nest on the Seward Peninsula in western Alaska and in coastal Russia (Lewis et al., 2021; Schmutz & Kondratyev, 1995). Emperor geese typically delay breeding until they have reached 3–4 years old (Schmutz, 2000), and often forgo nesting between breeding attempts (Hupp et al., 2006; Petersen, 1992b). The population of emperor geese declined drastically in the mid‐1980s, prompting harvest closures that lasted 3 decades (Pacific Flyway Council, 2016). A limited fall–winter harvest season and unrestricted spring–summer subsistence harvest were reopened in 2017 after population indices exceeded management objectives (Alaska Migratory Bird Co‐Management Council, 2016; Pacific Flyway Council, 2016). However, population growth has since slowed and indices fell below the threshold at which harvest closures must be considered in 2024 (U.S. Fish and Wildlife Service, 2024). Detailed studies of the ecology of emperor geese are needed to understand drivers of population change and potential responses to environmental change throughout their range.

The highest densities of emperor goose nests on the Yukon–Kuskokwim Delta are within 20 km of the Bering Sea coast (Saalfeld et al., 2017). Emperor geese generally nest near ponds and in areas with high cover of dwarf shrubs (e.g., *Salix* spp., *Empetrum nigrum*; Eisenhauer & Kirkpatrick, 1977; Petersen, 1990). Characteristics of nest sites, such as elevation, have been shown to vary with the timing of snow melt (Petersen, 1990). The primary causes of nest failure for emperor geese on the Yukon–Kuskokwim Delta are predation and flooding (Eisenhauer & Kirkpatrick, 1977; Thompson et al., 2023). The primary predators of emperor goose nests on the Yukon–Kuskokwim Delta are Arctic foxes (*Vulpes lagopus*), glaucous gulls (*Larus hyperboreus*), and parasitic jaegers (*Stercorarius parasiticus*; Eisenhauer & Kirkpatrick, 1977; Mickelson, 1975; Petersen, 1992a). Characteristics of nest sites, such as cover of tall dead vegetation, have been shown to affect survival of emperor goose nests (Petersen, 1990).

Our study took place near the Manokinak River (61.21° N, 165.10° W) on the Yukon–Kuskokwim Delta. The study area is generally low elevation (<5 m above the sea level) with many small, shallow freshwater ponds and tidal sloughs. Vegetation communities on the coastal region of the Yukon–Kuskokwim Delta, which encompasses our study area, vary with fine‐scale changes in geomorphology (Jorgenson, 2000). The dominant vegetation ecotypes in the study area, as described by Jorgenson (2000), consisted of lowland wet graminoid‐shrub meadows, lowland wet sedge meadows, coastal brackish wet graminoid‐shrub meadows, and coastal saline wet sedge meadows (Jorgenson & Roth, 2010). Within ecotypes, the landscape varies substantially in the vegetation structure, number and size of ponds, and density of nesting birds.

We systematically searched a ~23 km^2^ study area for all emperor goose nests from 2000 to 2017. Timing of initial nest searches was variable among years, but typically took place during egg laying or early incubation. Once nests were located, we recorded UTM coordinates using handheld GPS receivers (≤5 m accuracy) and candled or floated eggs to determine incubation stage (Weller, 1956; Westerskov, 1950). We used egg ages obtained from floating and candling to estimate hatch dates for nests when hatch was not directly observed. We captured adult female emperor geese on nests using bow net traps (Salyer, 1962) and mist nets (Bacon & Evrard, 1990) on or near their predicted hatch date. We also captured females during remigial molt by herding family groups into corral traps (Owen, 1980). These females captured during banding drives did not enter our sample until they were encountered on a nest in subsequent years. We banded captured females with a U.S. Geological Survey (USGS) metal leg band on one leg and a plastic tarsal band engraved with a 3‐character alphanumeric code on the other leg. In subsequent years, we resighted marked females on nests using spotting scopes or binoculars in the early years of the study period (2000–2010), and digital cameras in the later years of the study (2010–2017). Marked individuals were recaptured on the nest if their plastic tarsal band was worn or missing, or if additional data needed to be collected (e.g., blood samples). We revisited nests at irregular intervals (typically once per week) until a fate could be determined (hatched, preyed upon, abandoned, etc.), although crews often left field camps shortly after peak hatch to reduce disturbance, so not all nests were monitored until a fate could be determined in all years. Nests were considered successful if pipped eggs, goslings, or eggshell fragments with intact detached membranes were observed in the nest. We searched the study area from 1994 to 1998 but did not include these years in our analyses due to inconsistent data collection efforts (Thompson et al., 2023); therefore, emperor geese that were banded prior to 2000 entered our sample on the first occasion they were encountered on a nest between 2000 and 2017. We calculated the Euclidean distances (m) between nest locations for individuals observed in at least two consecutive years (hereafter, dispersal distance) using UTM nest coordinates, and only retained consecutive encounters for which dispersal distance could be calculated in our analyses. All data were collected under approved U.S. Geological Survey Institutional Animal Care and Use Committee protocols and authorized under U.S. Geological Survey federal bird banding permit number 20022. The data used in this study can be found in Thompson and Uher‐Koch (2024).

### 2.2 Statistical analyses

We fit two hierarchical generalized linear mixed models in a Bayesian framework to test our predictions. Our first model predicted dispersal distance between nests of individuals encountered in consecutive years as a function of previous nest fate (successful or failed), spring timing, and major flooding events, while our second model predicted daily survival probability of nests as a function of previous nest fate, dispersal distance, and their interaction. Descriptions of models and computational details are included below.

#### 2.2.1 Nest‐site fidelity model

We modeled dispersal distance of individual *i* in year *j* ($d_{i,j}$) on the log scale using a location‐scale *t*‐distribution (Plummer, 2017):

$$\log\left(d_{i,j}\right)∼t\left(g_{i,j},\sigma_{k}^{2},\kappa_{k}\right),$$

where $g_{i,j}$ is the expected dispersal distance of individual *i* in year *j* on the log scale, $\sigma_{k}^{2}$ is residual variation in dispersal distance on the log scale for previous nest fate *k*, and $\kappa_{k}$ is the degrees of freedom of the *t*‐distribution for previous nest fate *k*. We modeled the expected dispersal distance as a linear function of nest fate in the previous year ($f_{i,j-1}$; 0 for successful nests and 1 for failed nests), major flooding events that affected a large proportion of the study area and caused significant nest failure ($w_{j-1}$; 2010 and 2013; Thompson et al., 2023), an index of spring timing ($s_{j}$), and random individual ($\epsilon_{i}$) and annual ($\epsilon_{j}$) variation which we assumed was normally distributed on the log scale. We indexed spring timing using the early‐late index described by Kellett and Alisauskas (2019), which we calculated by subtracting the long‐term average mean nest initiation date from the mean nest initiation date for a given year using reported mean nest initiation dates for the entire study period from Thompson et al. (2023). The model took the form:

$$\begin{array}{l} g_{i,j}=\alpha_{j}+\beta_{f}f_{i,j-1}+\beta_{f\times w}f_{i,j-1}w_{j-1}+\epsilon_{\mathrm{ind},i},\\ \alpha_{j}=\mu +\eta_{s}s_{j}+\eta_{w}w_{j-1}+\epsilon_{\mathrm{yr},j},\\ \epsilon_{\mathrm{ind},i}∼\text{normal}\left(0,\varsigma_{\mathrm{ind},d}^{2}\right),\\ \epsilon_{\mathrm{yr},j}∼\text{normal}\left(0,\varsigma_{\mathrm{yr},d}^{2}\right), \end{array}$$

where $\alpha_{j}$ is the mean dispersal distance for year *j* on the log scale, $\beta_{f}$ is the regression coefficient for previous nest fate, $\beta_{f\times w}$ is the regression coefficient for the interaction between previous nest fate and major flooding event in the previous year, μ is the among‐year mean dispersal distance on the log scale, $\eta_{s}$ is the regression coefficient for spring timing, $\eta_{w}$ is the regression coefficient for major flooding events, $\varsigma_{\mathrm{ind},d}^{2}$ is the among‐individual variance in dispersal distance on the log scale, and $\varsigma_{\mathrm{yr},d}^{2}$ is the residual among‐year variance in dispersal distance on the log scale. Previous nest fates were missing for some encounters of individuals in some years (see *Study species and data collection*), although these encounters still contained information about dispersal distance. Therefore, rather than censoring these encounters, we included a missing data model to estimate previous nest fates for encounters where it was unknown (n=91; Hobbs & Hooten, 2015). We estimated previous nest fates as Bernoulli trials where the success probability was derived from the product of nest age‐ (days; a) and year‐specific daily nest survival probabilities ($\phi_{a,j-1}$) from the age on the day the nest was last checked ($m_{i,j-1}$) through 28 days, which is the average exposure period for an emperor goose nest assuming a 24‐day incubation period and a 4‐day laying period for a 5‐egg clutch (Eisenhauer & Kirkpatrick, 1977; Thompson et al., 2023):

$$f_{i,j-1}∼\text{Bernoulli}\left(1-\prod_{a=m_{i,j-1}}^{28}\phi_{a,j-1}\right).$$

We parameterized informative beta‐distributed priors for age‐ and year‐ specific daily nest survival probabilities using estimates from the Manokinak River study area reported by Thompson et al. (2023). We used the mean age‐specific daily nest survival probabilities across years for 2005 because Thompson et al. (2023) do not provide estimates for that year. We used vague prior distributions for the remaining parameters of the nest‐site fidelity model (Table 1).

**TABLE 1:** Prior distributions for parameters of the nest‐site fidelity model.

Parameter	Prior distribution
μ	normal(0,100)
$\beta_{f}$	normal(0,100)
$\beta_{f\times w}$	normal(0,100)
$\eta_{w}$	normal(0,100)
$\eta_{s}$	normal(0,100)
$\varsigma_{\mathrm{ind},d}$	uniform(0,3)
$\varsigma_{\mathrm{yr},d}$	uniform(0,3)
$\sigma_{k}$	uniform(0,3)
$\kappa_{k}$	gamma(1,1)

*Note*: Parameters include among‐year mean dispersal distance on the log scale (μ), regression coefficients for previous nest fate ($\beta_{f}$), major flooding events ($\eta_{w}$), an interaction between previous nest fate and major flooding events ($\beta_{f\times w}$), and spring timing ($\eta_{s}$), among‐individual standard deviation of dispersal distance on the log scale ($\varsigma_{\mathrm{ind},d}$), among‐year standard deviation of dispersal distance on the log scale ($\varsigma_{\mathrm{yr},d}$), residual standard deviation of dispersal distance on the log scale for each previous fate ($\sigma_{k}$), and degrees of freedom for each previous fate ($\kappa_{k}$). Informative prior distributions for parameters of the missing data model are described in the text and are not shown here.

#### 2.2.2 Nest survival model

We used nest monitoring data to generate exposure intervals for each nest following Dinsmore et al. (2002) and Thompson et al. (2023). We censored all nests where the response (nest fate) was unknown (*n* = 92), which included all nests in 2005 (*n* = 42). We modeled the nest fate of individual *i* in year *j* during exposure interval h ($y_{i,j,h}$) as Bernoulli distributed:

$$y_{i,j,h}∼\text{Bernoulli}\left({\phi_{i,j}}^{l_{i,j,h}}\right),$$

where $\phi_{i,j}$ is the daily nest survival probability of individual *i* in year *j* and $l_{i,j,h}$ is the length (in days) of exposure interval *h* for individual *i* in year *j*. We modeled $\phi_{i,j}$ on the logit scale as a linear function of previous nest fate, dispersal distance, their interaction, and random individual ($\varepsilon_{\mathrm{ind},i}$) and annual variation as follows:

$$\begin{array}{c} \begin{array}{l} \text{logit}\left(\phi_{i,j}\right)=\pi_{j}+\gamma_{d}d_{i,j}+\gamma_{f}f_{i,j-1}+\gamma_{d\times f}d_{i,j}f_{i,j-1}+\varepsilon_{\mathrm{ind},i},\\ \pi_{j}∼\text{normal}\left(\delta ,\varsigma_{\mathrm{yr},\phi }^{2}\right),\\ \varepsilon_{\mathrm{ind},i}∼\text{normal}\left(0,\varsigma_{\mathrm{ind},\phi }^{2}\right), \end{array} \end{array}$$

where $\pi_{j}$ is the mean daily nest survival probability on the logit scale in year *j* for individuals with a successful nest in the previous year, $\gamma_{d}$ is the regression coefficient for dispersal distance of individuals with a successful nest in the previous year, $\gamma_{f}$ is the regression coefficient for previous nest fate, $\gamma_{d\times f}$ is the regression coefficient for the interaction between dispersal distance and previous nest fate, δ is the mean daily nest survival probability across years on the logit scale for individuals with successful nests in the previous year, $\varsigma_{\mathrm{yr},\phi }^{2}$ is the among‐year variance in daily nest survival probability on the logit scale, and $\varsigma_{\mathrm{ind},\phi }^{2}$ is the among‐individual variance in daily nest survival probability on the logit scale. We estimated missing previous nest fates (*n* = 78) using the same methods as the nest‐site fidelity model. We derived estimates of nest survival probability by exponentiating estimated daily nest survival probabilities to the power of 28. We used vague prior distributions for all parameters of the nest survival model (Table 2).

**TABLE 2:** Prior distributions for parameters of the nest survival model.

Parameter	Prior distribution
δ	logistic(0,1)
$\gamma_{d}$	normal(0,100)
$\gamma_{f}$	normal(0,100)
$\gamma_{d\times f}$	normal(0,100)
$\varsigma_{\mathrm{ind},\phi }$	uniform(0,3)
$\varsigma_{\mathrm{yr},\phi }$	uniform(0,3)

*Note*: Parameters include the mean daily nest survival probability across years on the logit scale (δ), regression coefficients for dispersal distance ($\gamma_{d}$), previous nest fate ($\gamma_{f}$), and an interaction between dispersal distance and previous nest fate ($\gamma_{d\times f}$), among‐individual standard deviation of daily nest survival probability on the logit scale ($\varsigma_{\mathrm{ind},\phi }$), and the among‐year standard deviation of daily‐nest survival probability on the logit scale ($\varsigma_{\mathrm{yr},\phi }$). Informative prior distributions for parameters of the missing data model are described in the text and are not shown here.

#### 2.2.3 Interpretation and computational details

We fit both models in JAGS using the “jagsUI” package in R (Kellner, 2016; Plummer, 2003; R Core Team, 2022). For each model, we drew inference from three Markov chain Monte Carlo (MCMC) chains of 80,000 iterations, where we discarded the first 40,000 iterations as burn‐in. We used a noncentered parameterization for zero‐centered random effects ($\epsilon_{\mathrm{ind},i},\epsilon_{\mathrm{yr},j},\;\text{and}\;\varepsilon_{\mathrm{ind},i}$; Betancourt & Girolami, 2015) to improve the mixing of MCMC chains. We assessed convergence of MCMC chains by inspecting trace plots of parameters for proper mixing and by evaluating Gelman–Rubin test statistics (Brooks & Gelman, 1998). We assessed model fit for each model using posterior predictive checks (Conn et al., 2018). Specifically, we calculated Bayesian *p*‐values for the median and standard deviation (nest‐site fidelity model) and the mean (nest survival model) of the observed data and data simulated using the models. We interpreted Bayesian *p*‐values >.90 and <.10 as evidence for lack of fit (Conn et al., 2018; Hobbs & Hooten, 2015). After checking for convergence and model fit, we summarized posterior distributions of each parameter with the median and 95% highest posterior density interval (95% HPDI). For regression coefficients, we report the proportion of the posterior samples that have the same sign as the mean (ρ) where we interpreted ρ as the probability that the parameter is greater than (or less than) zero given our model assumptions.

## 3 RESULTS

**FIGURE 2:**
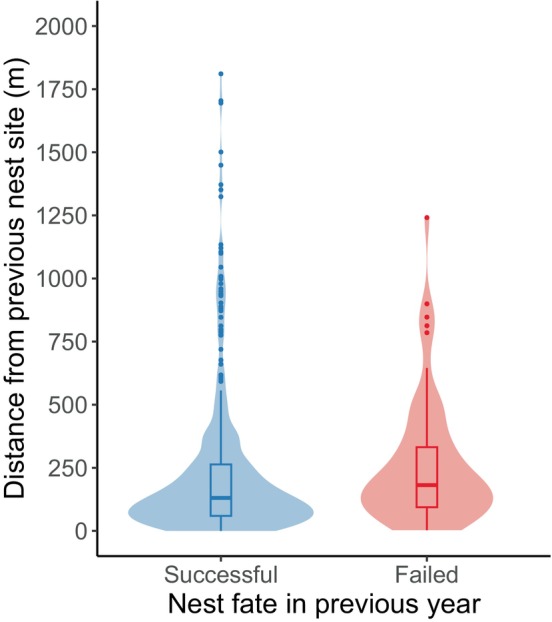
Distributions of observed nest‐site dispersal distances (m) for emperor geese that nested successfully in the previous year and those that failed their nesting attempt in the previous year. Dispersal distances over 2000 m are not shown (*n* = 12 previously successful nests, maximum = 4236 m). Data were collected near the Manokinak River on the Yukon–Kuskokwim Delta in western Alaska from 2000 to 2017.

Our sample consisted of 602 pairs of consecutive encounters of 245 individually marked emperor geese from 2000 to 2017 (Thompson & Uher‐Koch, 2024). Of these, 446 were encounters following a successful nesting attempt, 65 were encounters following a failed nesting attempt, and 91 were encounters where the previous nest fate was unknown. The number of consecutive encounters of unique individuals ranged from 2 to 12. Dispersal distances were highly skewed for both geese that nested successfully and geese that failed in the previous year (Figure 2). The shortest dispersal distance observed between consecutive nesting attempts was 0 m, while the greatest observed dispersal distance was 4236 m.

**FIGURE 3:**
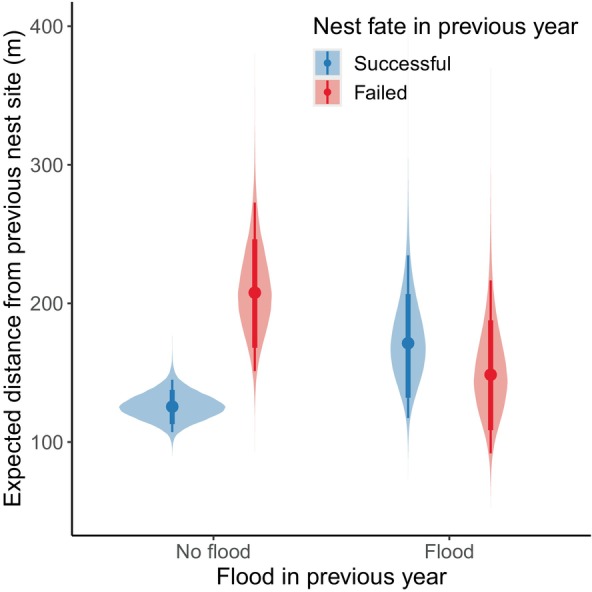
Posterior distributions of expected dispersal distance (m) for emperor geese that nested successfully the previous year (blue) and individuals that failed their nesting attempt in the previous year (red), and following years with and without major flooding events. Predictions account for among‐year and among‐individual variation in expected dispersal distance. Points are posterior medians, thick lines are 80% highest posterior density limits, and thin lines are 95% highest posterior density limits. Data were collected near the Manokinak River on the Yukon–Kuskokwim Delta in western Alaska from 2000 to 2017.

Our nest‐site fidelity model included all 602 pairs of encounters. Inspection of traceplots and Gelman–Rubin test statistics indicated convergence of the MCMC chains for parameters of the nest‐site fidelity model, and our posterior predictive checks indicated adequate model fit for the median (*p* = .594) and standard deviation (*p* = .628). Expected dispersal distance for geese that nested successfully in the previous year was 125.5 m (95% HPDI: 107.1–144.9; Figure 3). The expected dispersal distance was greater for geese that failed their nesting attempt in the previous year (207.7 m, 95% HPDI: 151.1–272.7), which was supported by our regression coefficient ($\beta_{f}=0.506$, 95% HPDI: 0.206–0.795, ρ=0.999; Figure 3). We found strong evidence that expected dispersal distance was slightly greater following years of major flooding events for individuals that nested successfully ($\eta_{w}=0.310$, 95% HPDI: −0.050 to 0.662, ρ=0.959; Figure 3). However, our interaction term indicated that this was not the case for geese that failed their nesting attempt in the previous year ($\beta_{f\times w}=-0.647$, 95% HPDI: −1.205 to −0.084, ρ=0.988), with expected dispersal distance being lower for individuals that failed their nesting attempt in a year of a major flooding event than for individuals that failed in a year without a major flooding event (Figure 3). We did not find evidence that expected dispersal distance was influenced by our index of spring timing ($\eta_{s}=-0.002$, 95% HPDI: −0.019 to 0.015, ρ=0.597). There was substantial among‐individual heterogeneity in dispersal distance ($\varsigma_{\mathrm{ind},d}=0.683$, 95% HPDI: 0.536–0.831), but little among‐year variation ($\varsigma_{\mathrm{yr},d}=0.080$, 95% HPDI: 0.000–0.213).

**FIGURE 4:**
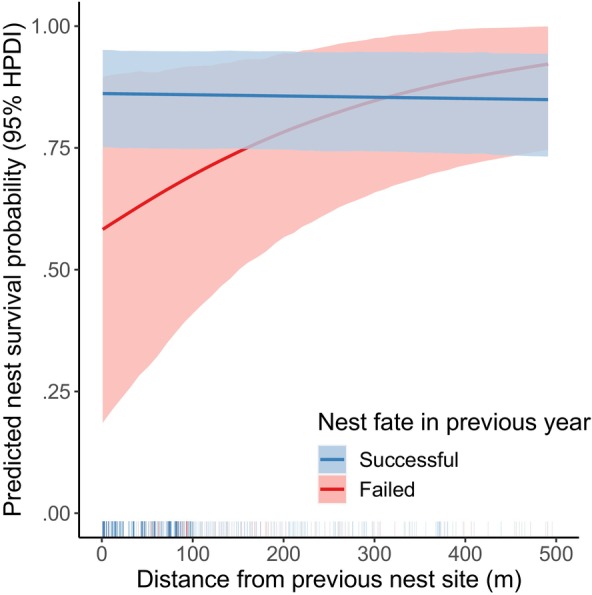
Predicted relationship between nest survival probability (with 95% highest posterior density limits) and nest‐site dispersal distance (m) for emperor geese that nested successfully on their previous nesting attempt (blue) and failed their previous nesting attempt (red). Nest survival probability was derived by exponentiating estimated daily survival probabilities to the power of 28. Predictions account for among‐year and among‐individual variation in daily nest survival probability. Rug shows the observed dispersal distances for each fate and does not include nests where previous nest fate was not observed. Data were collected near the Manokinak River on the Yukon–Kuskokwim Delta in western Alaska from 2000 to 2017.

Our nest survival model included 510 nests with known fates. Inspection of traceplots and Gelman–Rubin test statistics indicated convergence of MCMC chains for parameters of the nest survival model, and posterior predictive checks indicated adequate model fit for the mean (*p* = .694). Mean daily nest survival probability for geese with successful nests in the previous year was high (0.994, 95% HPDI: 0.989–0.998) and was not affected by previous nest fate ($\gamma_{f}=-0.115$, 95% HDPI: −0.989 to 0.941, ρ=0.593). We found weak support for a slight negative relationship between dispersal distance and daily nest survival probability for geese that nested successfully in the previous year ($\gamma_{d}=-0.0002$, 95% HPDI: −0.001 to 0.000, ρ=0.791). We found strong support for an interaction between dispersal distance and previous nest fate ($\gamma_{d\times f}=0.004$, 95% HPDI: 0.000–0.009, ρ=0.975), indicating a positive relationship between dispersal distance and daily nest survival probability for geese that failed their nesting attempt in the previous year (Figure 4). There was little among‐individual variation in daily nest survival probability ($\varsigma_{\mathrm{ind},\phi }=0.176$, 95% HPDI: 0.000–0.508), but substantial among‐year variation ($\varsigma_{\mathrm{yr},\phi }=1.177$, 95% HPDI: 0.570–2.034).

## 4 DISCUSSION

The win‐stay lose‐switch strategy predicts that an individual will be more likely to return to a site if they were previously successful at that site, and more likely to disperse if they were not successful. We found that fine‐scale nest‐site dispersal decisions of emperor geese were affected by nest fate in the previous year consistent with the win‐stay lose‐switch strategy, where individuals that failed their nesting attempt in the previous year disperse further on average than individuals that nested successfully in the previous year. Our results also indicate that the win‐stay lose‐switch strategy is adaptive for emperor geese in that dispersing a greater distance in years following a failed nesting attempt led to a higher probability of nesting successfully. Contrary to our prediction, we found that fine‐scale nest‐site dispersal decisions were not influenced by timing of spring on the study area. However, we found that individuals dispersed greater distances on average following years of major flooding events, suggesting that extreme weather events contribute to dispersal decisions of emperor geese.

The win‐stay lose‐switch strategy is expected when habitat quality varies spatially but is predictable through time (Switzer, 1993). Quality of nesting habitat for birds is related to risk of nest failure, which is typically due to predation. Predictable spatial variation in the risk of predation might favor the win‐stay lose‐switch strategy for emperor geese. Predictable spatial variation in predation pressure could result from consistent distribution of predators on the landscape, including glaucous gulls that typically nest in colonies on large lakes (Strang, 1976). The distribution of Arctic foxes on the landscape is likely not consistent due to their large home ranges (Anthony, 1997), and predation pressure from Arctic foxes may not be predictable because abundance of Arctic foxes on the Yukon–Kuskokwim Delta varies annually (Fischer et al., 2017). In addition to consistent distribution of predators, predictable spatial variation in the risk of predation may occur if patches of certain habitat types consistently minimize the risk of predation (Lecomte, Careau, et al., 2008; Stickney, 1991; Strang, 1976), regardless of predator abundance. For example, Lecomte, Careau, et al. (2008) found that Arctic foxes were less efficient at foraging on greater snow goose (*Anser caerulescens atlanticus*) nests in wetland complexes, and that nest success was generally higher in those patches (Lecomte, Gauthier, et al., 2008). Observer activity, such as nest visits and nest trapping, may also cause nest failure and subsequently influence nest‐site fidelity decisions of emperor geese regardless of the environment (Thompson et al., 2023), although we suspect that the influence of observer effects was minimal in our study as nests were visited infrequently and birds were trapped near or on the predicted hatch date of their nest.

Evidence for the win‐stay lose‐switch strategy is common among birds, but evidence of its adaptive significance is not consistent among species and often depends on the vital rate considered (e.g., Lindberg & Sedinger, 1997; Norton & DuVal, 2023). Our results support the conclusion that the win‐stay lose‐switch strategy is adaptive for emperor geese in that it leads to higher probability of nest survival (Figure 4). Nesting further away from a previous nest site may be associated with changes in nest‐site characteristics that could reduce the risk of predation or flooding (e.g., vegetation cover, density of nesting geese, elevation), although we were not able to test this in our study. A comprehensive understanding of the adaptive significance of the win‐stay lose‐switch strategy for emperor geese and other species would require future studies that consider dispersal distance and changes in nest‐site characteristics simultaneously (Benvenuti et al., 2018), as well as additional fitness measures.

While our results suggest that fine‐scale nest‐site dispersal decisions of emperor geese follow the win‐stay lose‐switch strategy, we emphasize that the difference between expected dispersal distance of individuals that nested successfully in the previous year and those that failed their nesting attempt in the previous year was small (Figure 3). We were not able to account for imperfect detection or skipped breeding due to limitations in our capture‐mark‐reencounter data, therefore our analyses relied solely on paired encounters of individuals observed on nests in the study area in consecutive years. Previous nest fate of an individual might influence their availability for detection in subsequent years by affecting the probability of temporarily or permanently dispersing out of the study area or breeding probability (Souchay et al., 2014). Furthermore, experience in years prior to the year before an observation may influence dispersal distance, although we did not attempt to test this because we may not have observed all previous breeding attempts, particularly for birds banded early in the study period. Lastly, studies of dispersal in birds are sensitive to the scale of observations (e.g., the size of the study area, Van Noordwijk, 1995), which could result in reduced detection of large movements. We suspect that these limitations might cause our estimates of expected dispersal distance for geese that failed their previous nesting attempt to be conservative.

The reproductive benefits of nesting early for geese may cause individuals to initiate a nest when the landscape is still largely covered by snow during late springs (Petersen, 1990). This could make dispersal more likely in late springs as previous nest sites may not be available (Lecomte, Gauthier, et al., 2008). Surprisingly, we found that dispersal distance of emperor geese was unaffected by spring timing. Emperor geese that initiate nests early generally nest on ridges or areas with more microtopographic relief than those that initiate nests late in years of prolonged snow cover (Petersen, 1990). Furthermore, the timing of nest initiation for individual emperor geese is similar among years (Petersen, 1992b). We hypothesize that similar dispersal distances in early and late springs may be caused by repeated patterns in nest‐site selection of individuals related to consistent among‐individual variation in nest initiation dates. Nest initiation date may be related to individual quality in geese (Lohman et al., 2021), suggesting that variation in quality among individuals could contribute to the observed among‐individual variation in expected dispersal distance. Alternatively, because emperor geese tend to nest in areas with more microtopographic relief (Petersen, 1990), nest sites for emperor geese simply may not be limited in late springs as these areas often thaw and drain of meltwater before lower elevation areas.

Major tidal flooding events on the coastal zone of the Yukon–Kuskokwim Delta are primarily caused by storm surges (Terenzi et al., 2014), which are uncommon during the spring and early summer months when birds are nesting. Major flooding events on the study area contributed to years of low nest survival for emperor geese at the Manokinak River in 2010 and 2013 (Thompson et al., 2023), but only led to an increase in expected dispersal distance the following year for individuals that nested successfully (Figure 3). We suspect that although the flooding events may not have caused nest failure for these individuals, it could have caused reproductive failure by flooding recently hatched nests (e.g., within 24 h of hatch). Additionally, reproductive success of conspecifics could influence dispersal decisions in birds (Citta & Lindberg, 2007; Doligez et al., 2002; Rioux et al., 2011; Van Dellen & Sedinger, 2022), which might explain this result if successful individuals acquired information about poor reproductive success in flooded areas.

We were surprised that expected dispersal distance was slightly lower for individuals that failed their nesting attempt during a flood year than for individuals that failed their nesting attempt during a year without a flood (Figure 3). Flooding events occurred 5 days prior to the mean hatch date in 2010 (16 June), and on the mean hatch date in 2013 (29 June). Therefore, individuals that failed their nesting attempts were likely close to hatching their nest at the time of the flood. If the risk of flooding at a given nest site is unpredictable and flooded sites are otherwise high‐quality nest sites (e.g., low predation pressure, access to resources), then fidelity to those nest sites may be adaptive regardless of whether individuals failed due to floods, as dispersal may lead to predictable fitness consequences (Switzer, 1993).

The expected nest‐site dispersal distance of emperor geese in our study was small (i.e., less than 300 m) despite previous nest fate. This could suggest that emperor geese exhibit consistent fidelity (i.e., always‐stay strategy) to breeding sites at a larger scale, such as the patch scale, as has been shown for other bird species (Byrne et al., 2022; Gerber et al., 2019), although we did not examine this in our study because we were unable to delineate discrete habitat patches in our study area post hoc. Breeding‐site fidelity at the patch level may be driven by a variety of factors, including spatial and temporal variation in patch quality and public information on performance of conspecifics within a patch (Naves et al., 2006). Future work on patch‐scale fidelity that incorporates variation in patch quality and public information would help to further understand observed patterns in nest‐site fidelity of emperor geese.

Our study adds to a growing body of work focused on identifying the causes and consequences of nest‐site fidelity for birds and the effects of environmental variation on dispersal decisions. Ongoing climate change has led to an increase in variability of environmental conditions in northern latitudes such as the Arctic (Schmidt et al., 2023). While our results provide evidence for use of the win‐stay lose‐switch strategy in dispersal decisions of emperor geese and its adaptive significance, the adaptive benefits of the win‐stay lose‐switch strategy may be diminished if increased variability in environmental conditions on breeding areas leads to a decrease in predictability of reproductive outcomes (Kloskowski, 2021; Merkle et al., 2022). Examining potential changes in nest‐site fidelity behaviors due to climate change could be a valuable area for future research, particularly for species nesting in high northern latitudes like emperor geese.

## AUTHOR CONTRIBUTIONS

**Jordan M. Thompson:** Conceptualization (equal); data curation (equal); formal analysis (lead); visualization (lead); writing – original draft (lead); writing – review and editing (equal). **Brian D. Uher‐Koch:** Conceptualization (equal); data curation (equal); investigation (supporting); writing – review and editing (equal). **Bryan L. Daniels:** Conceptualization (equal); funding acquisition (supporting); writing – review and editing (equal). **Thomas V. Riecke:** Formal analysis (supporting); visualization (supporting); writing – review and editing (equal). **Joel A. Schmutz:** Conceptualization (equal); funding acquisition (lead); investigation (lead); project administration (lead). **Benjamin S. Sedinger:** Conceptualization (equal); formal analysis (supporting); funding acquisition (supporting); writing – review and editing (equal).

## CONFLICT OF INTEREST STATEMENT

The authors have no competing interests to declare.

## Data Availability

Data and code for this analysis are available in Thompson and Uher‐Koch (2024).
